# On the Pupil, and Accidents Attending It

**Published:** 1817-10

**Authors:** N. Littleton


*For the London Medical and Physical Journal.*

### *On the Pupil, and Accidents attending it;* by N. Littleton, Esq.

COMMUNICATIONS that to the superficial glance appear trifling, may, from the experience of utility in practice, be thought deserving more attention. If what I am about to mention be one of these, it may, perhaps, be admitted into your Journal.

A little girl, in play, pushed the eye of a wooden doll into the meatus auditorius externus. The eye was made of coarse porcelain, having its shortest or conjugate axis about three quarters of the diameter of the meatus, and, by its turning a little obliquely to its long axis, it readily stuck in the passage, and so far in, that it could be just seen when the external ear was pulled so as to render the meatus less tortuous.

The bead, thus situated, I could not remove with such forceps, curved probe, &c. as were at hand; and, as the touching of the tender lining of the meatus also caused the child to cry, such attempts were given over. I afterwards injected some warm water, in order to remove any cerumen that might prevent the bead from being so clearly seen. At first I injected it slowly, and gradually used more force, as I saw that it did not make the bead to recede further, which probably arose from its already resting on the membrana tympani. At last I forced the water in with as much power as a urethral syringe would allow, and soon observed that the bead moved outwards, so as readily to be removed with a probe.

Having thus succeeded, I was desirous of knowing the greatest retropulsive force of fluids thus injected. I therefore took a goose-quill, two inches long, and having plugged one end of it, introduced a pebble of such a size as to stick slightly at the bottom of the quill. With the syringe I found it was readily removed, as was also any other pebble thus slightly stuck, provided that it was not so perfectly fitted to the interior of the quill as to prevent the water from passing behind it.

 Now

Now the great power which fluids have when thus used in dislodging such bodies, and to what extent we may trust to injection, is what I wish to make more known, for even Celsus mentions *syringing* with the same intention, and also concussion of the body, in the following ingenious manner:—"Tabula quoque collocatur media, inhærens capitibus utrinque pendentibus, superque eam homo deligatur, in id latus versus, cujus auris eo modo laborat, sic, ut extra tabulam non emineat: tum mulleo caput tabulæ, quod à pedibus est, feritur; atque ita concussa aure, id quod inest, excidit."

---

In the 36th volume of your valuable Journal, I have mentioned some facts relating to the size of the pupil. With regard to my remarks on the effects of convulsion, it becomes my duty to make early mention of a case which I have lately attended to.

M. H., æt. about 40, of a delicate constitution, has been subject to fits twenty-three or twenty-four years. I was called to her a few nights since, when affected with violent pain in the bowels, accompanied with vomiting and purging. Judging, by pressure on the abdomen, by the pulse, &c. that the pain was spasmodic, I gave her tinct. opii gtt. 100. In a few minutes, on asking her concerning the pain, I found her incapable of answering, lying with her countenance composed, eyes closed, and her breathing so easy as hardly to be perceptible. Soon after, she became convulsed, struggled violently, and shrieked loudly, which recurred at intervals for an hour, during which I had frequent opportunities of examining the pupil, as sometimes the eyes were open during the convulsion, and occasionally I opened them during her struggling purposely to examine the irides. Towards the termination of the convulsion, she talked incoherently, and gave incoherent answers, and it finally ended with sorrowful crying.

During the convulsion, the pupil was as small as I should expect it to be during sleep, but, in the intervals between the returns of spasm, the pupil enlarged on removing the candle from the eyes just as much as it did after the convulsion had entirely ceased, and she had become sensible and reasonable. I have since seen her similarly attacked with pain, when I gave her the opiate as before, and she became similarly convulsed, when I had an opportunity of again observing the same state of the pupil as before.

Now, this state of the pupil in convulsion is what I have never before observed, although I have particularly attended to it for some years past. On the contrary, I had always observed its enlargement in all cases where there was much action of the voluntary muscles, except in one case of hydrocephalus, where there was an alternating action of the flexors and extensor muscles of the fore-arm, as mentioned vol. 36, page 267.

This case of convulsion will readily be considered as hysterical, especially when I add that there was no foaming at the mouth, no lividity of the face, no heaving at the sternum, or unnatural distortion of the countenance; but, during the most violent spasms, there was always a natural expression on the countenance, and the tonic spasm of the respiratory muscles soon ended in shrieking, the colour of the cheek being only suffused with redness without any lividity.

Therefore I think it must be admitted, that, in some cases of hysterical convulsion, the pupil, instead of being enlarged as usual, is, on the contrary, diminished in size, as in sleep, stupor, &c. It is right to remark, that I have many times, and in various females, observed the pupil enlarged in convulsion, which I considered hysterical, and where I had given much larger doses of tinct. opii.

In my last paper on the iris, when speaking of the effect of exhaustion, I unintentionally omitted to instance the state of the pupil in syncope from gradual loss of blood, &c. during which state of the body, when such syncope is complete, the pupil is as large as when affected by belladonna.

I shall refrain from mentioning any additional remarks on the iris at present, but hope to do so at some future period, as I have since been enabled to add to my former observations.

*Padstow; August* 6, 1817.

